# Epidemiology of subsequent bloodstream infections in the ICU

**DOI:** 10.1186/s13054-018-2148-0

**Published:** 2018-10-11

**Authors:** Niccolò Buetti, Elia Lo Priore, Rami Sommerstein, Andrew Atkinson, Andreas Kronenberg, Jonas Marschall, the Swiss Centre for Antibiotic resistance (ANRESIS), A. Burnens, A. Cherkaoui, V. Gaia, O. Dubuis, A. Egli, D. Koch, A. Kronenberg, S. Luyet, P. Nordmann, V. Perreten, J.-C. Piffaretti, G. Prod’hom, J. Schrenzel, S. L. Leib, A. F. Widmer, G. Zanetti, R. Zbinden

**Affiliations:** 1 Department of Infectious Diseases, University Hospital Bern, Bern, Switzerland; 2 Institute for Infectious Diseases, University of Bern, Bern, Switzerland

## Abstract

Subsequent bloodstream infections (sBSI) occur with a delay after removal of the intravascular catheter (IVC) whose tip revealed microbial growth. Here we describe the epidemiology of sBSI in the intensive care setting. Serratia marcescens, Staphylococcus aureus, Pseudomonas aeruginosa, and yeast were the pathogens most frequently associated with sBSI. In contrast, Enterococci were rarely found in sBSI.

**Electronic supplementary material:**

The online version of this article (10.1186/s13054-018-2148-0) contains supplementary material, which is available to authorized users.

## Letter

A recently published review on the management of catheter-related infection highlighted the clinical importance of a positive catheter culture without concomitant positive blood cultures in the ICU [1]. Recently, we conducted a nationwide, observational study on all positive intravascular catheter (IVC) tip cultures in Switzerland investigating subsequent bloodstream infections (i.e., bloodstream infection occurring after the catheter has been removed) with non-ICU and ICU data [2]. Interestingly, the studies investigating this topic reported either data from an individual hospital [1] or focused on single pathogens [3, 4]. Moreover, only one observational study studied the ICU population [5]. Based on the Swiss Antibiotic Resistance Surveillance System (ANRESIS), we aimed to describe the current epidemiology of culture-positive IVC tips without concurrent bacteremia in the ICU and to characterize bacteremia or fungemia occurring after catheter removal.

We conducted a nationwide surveillance study on all positive IVC tip cultures recovered in Swiss ICUs (36 hospitals) from 2008 to 2015. An IVC tip culture, which required IVC removal, was included in the analysis if at least one microorganism could be cultivated. We excluded data from patients with concurrent bacteremia and fungemia with the same microorganism identified 7 days before to 2 days after IVC removal (623 cases). Subsequent bloodstream infection (sBSI) was defined as isolating (from blood cultures performed > 2 days up to 7 days after IVC removal) the same microorganism as the one recovered from the IVC tip.

**Fig. 1:**
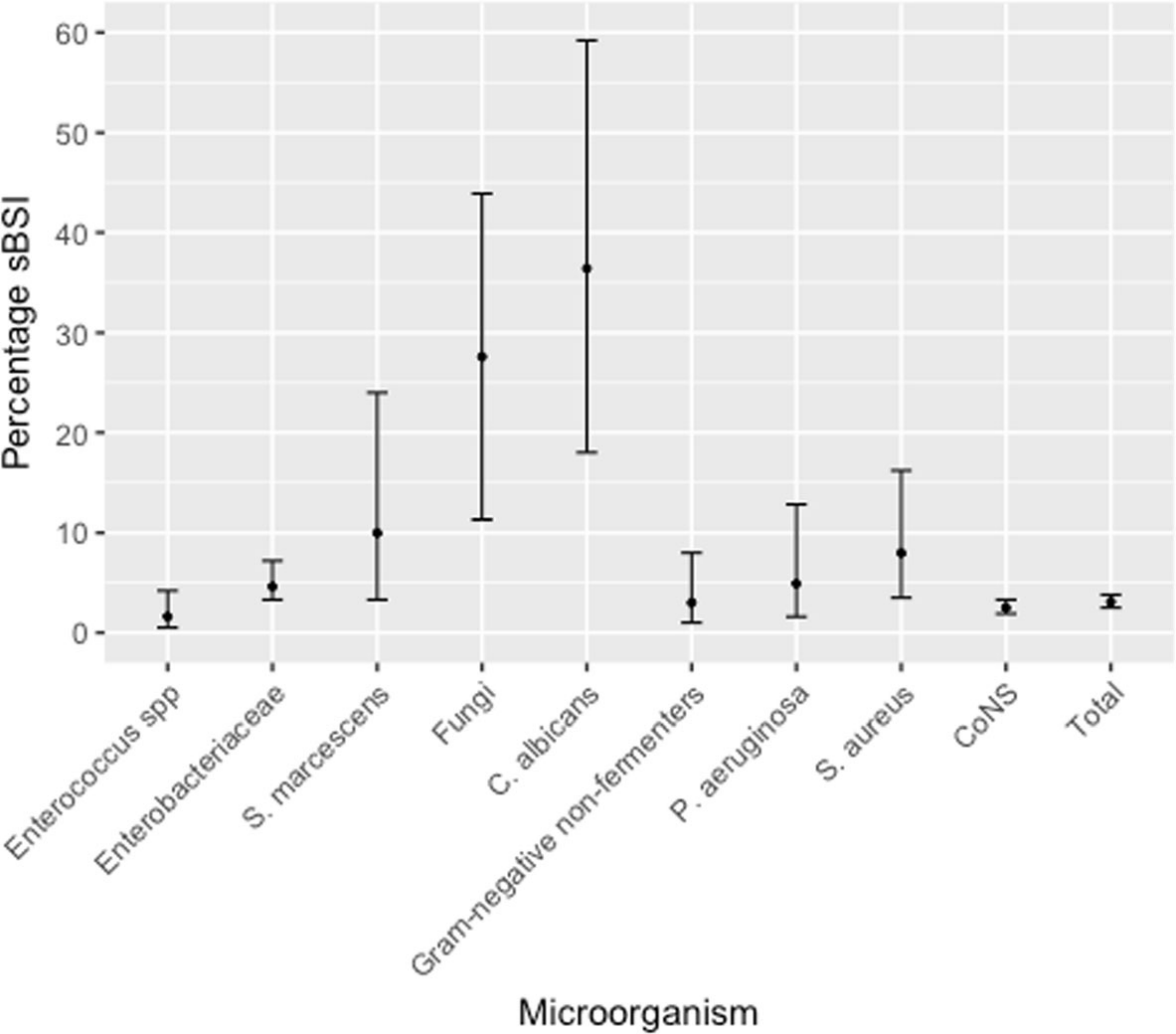
Proportion and confidence intervals of subsequent bloodstream infections (*sBSI*) in the ICU

Over the 8-year period, 2,941 positive IVC tip cultures without concurrent bacteremia were identified in ICUs. In 3.1% (92/2,941, 95% confidence interval 2.5–3.8) of removed catheters an sBSI was observed (Fig. 1). Among bacterial microorganisms, *Serratia marcescens* (4/40, 10%, 3.3–24), *Staphylococcus aureus* (7/88, 8.0%, 3.5–16.2) and *Pseudomonas aeruginosa* (4/81, 4.9%, 1.6–12.8) were the most frequently identified agents causing sBSI. Subsequent fungemia developed in 8/29 (27.6%, 11.3–43.9) IVC tips positive for fungi (Additional file 1: Table S1). Enterococci rarely caused sBSI (1.6%, 0.5–4.2).

To our knowledge, ours is the largest epidemiologic description of sBSI in this setting. Our findings highlight that particular attention should be paid if *Candida albicans*, *S. aureus*, *S. marcescens*, and *P. aeruginosa* are detected on an IVC tip. The presence of these four microorganisms is associated with a higher frequency of sBSI than other microorganisms and, therefore, a short treatment may need to be considered by intensive care physicians. In contrast, enterococci represented the lowest risk for sBSI and probably do not require specific antimicrobial therapy.

## Additional file

Additional file 1: **Table S1.** Microorganism distribution of positive catheter tip culture and sBSI in the ICU. (DOCX 16 kb)
