# Supplementary material for: Epidemiology of subsequent bloodstream infections in the ICU
Source: Crit Care. 2018 Oct 11;22:259. doi: 10.1186/s13054-018-2148-0 (PMC6180638; doi:10.1186/s13054-018-2148-0)
Supplement: Supplementary file 1 — Table S1. Microorganism distribution of positive catheter tip culture and sBSI in the ICU. (DOCX 16 kb) [file 13054_2018_2148_MOESM1_ESM.docx]

**Table S1 Microorganism distribution of positive catheter tip culture and sBSI in the ICU**

|  | Total* | sBSI | sBSI/pathogen isolated from catheter tip (95% CI) |
| --- | --- | --- | --- |
| *Enterococcus* spp | 252 | 4 | 1.6% (0.5-4.2) |
| Enterobacteriaceae | 412 | 19 | 4.6% (3.3-7.2) |
| *S. marcescens* | 40 | 4 | 10.0% (3.3-24) |
| Fungi | 29 | 8 | 27.6% (11.3-43.9) |
| *C. albicans* | 22 | 8 | 36.4% (18-59.2) |
| Gram-negative non-fermenters | 133 | 4 | 3.0% (1-8) |
| *P. aeruginosa* | 81 | 4 | 4.9% (1.6-12.8) |
| *S. aureus* | 88 | 7 | 8.0% (3.5-16.2) |
| CoNS¹ | 1925 | 49 | 2.5% (1.9-3.3) |
| Anaerobes | 1 | 0 | 0% |
| Other | 101 | 1 | 1% (0-6.2) |
|  |  |  |  |
| Total | 2941 | 92 | 3.1% (2.5-3.8) |

Note: CoNS: Coagulase-negative Staphylococci. spp: species.*Without episodes of bacteremia 7d before and 2d after catheter removal. CI: confidence interval. sBSI: subsequent bacteremia or fungemia. ¹ In 1360 positive catheter tips the CoNS subtype was not specified
